# The role of ferroptosis in esophageal cancer

**DOI:** 10.1186/s12935-022-02685-w

**Published:** 2022-08-23

**Authors:** Zimin Wang, Sikai Wu, Chengchu Zhu, Jianfei Shen

**Affiliations:** 1 Department of Thoracic Surgery, Taizhou Hospital of Zhejiang Province Affiliated to Wenzhou Medical University, Linhai, China; 2 Key Laboratory of Minimally Invasive Techniques & Rapid Rehabilitation of Digestive System Tumor of Zhejiang Province, Linhai, China

**Keywords:** Esophageal cancer, Ferroptosis, Cell death, Iron, Lipid peroxidation

## Abstract

Esophageal cancer is one of the most common cancers with high mortality rate around the world. Although the treatment strategy of this disease has made great progress, the prognosis of advanced patients is not ideal. Ferroptosis, a novel regulatory cell death model, that is different from traditional apoptosis and characterized by increased Fenton reaction mediated by intracellular free iron and lipid peroxidation of cell membrane. Ferroptosis has been proved to be closely linked to a variety of diseases, especially cancer. This review aims to summarize the core mechanism of ferroptosis in esophageal cancer, the regulation of ferroptosis signaling pathway and its current application. At the same time, we emphasize the potential and prospect of ferroptosis in the treatment of esophageal cancer. Collectively, targeting ferroptosis pathway may provide new insights into the diagnosis, treatment and prognosis of esophageal cancer.

## Introduction

Esophageal cancer (EC), a heterogeneous disease, can be broadly divided into esophageal squamous cell carcinoma (ESCC) and esophageal adenocarcinoma (EAC) [1]. According to statistics, the global incidence of EC in 2020 was about 604,000 cases, accompanied by 544,000 deaths [2]. At present, the effective means for treatment of EC mainly includes surgery, preoperative radiotherapy and chemotherapy or perioperative chemotherapy, immunotherapy and so on [3]. In recent years, great progress has been made in the immunotherapy of EC, but it is still not ideal for patients with advanced metastasis of EC. Unfortunately, many patients are diagnosed with EC when it is already diagnosed at advanced stage with distant metastases. Importantly, ESCC patients first diagnosed with distant organ metastases in a retrospective study had a particularly low survival rate, with a 6-month median survival [4]. Therefore, there is a critical need for alternative strategies for more effective treatment of EC.

Ferroptosis is a novel form of cell death and becomes the research hotspot in recent years [5]. It is a kind of non-apoptotic cell death characterized by accumulation of intracellular iron and reactive oxygen species (ROS). Ferroptotic cells display some special morphological changes, such as smaller mitochondria than normal cells, contraction of mitochondrial membrane, reduction or disappearance of mitochondrial crest, and rupture of outer membrane [6]. A vital pathway in ferroptosis is ROS-mediated lipid peroxidation. Several key ROS-related proteins such as glutathione peroxidase 4(GPX4) [7], cystine/glutamate transporter (system X_C_^−^) [5], lipoxygenase (LOX) [8], and nitrogen oxides (NO_X_) [9] regulate ferroptosis by influencing lipid ROS pathway. Induction of ferroptosis with chemical modulators, radiotherapy and immunotherapy has emerged as a promising anti-neoplastic therapy [10]. Encouragingly, emerging preclinical evidence suggests that inducing ferroptosis may have considerable potential for the treatment of ESCC. For instance, the level of DnaJ/Hsp40 homolog, subfamily B, member 6 (DNAJB6) in patients with EC is negatively correlated with lymph node metastasis [11]. Meanwhile, the overexpression of DNAJB6a can promote ferroptosis in ESCC through mechanisms that remain poorly defined [11]. Moreover, oridonin, a tetracyclic diterpenoid extracted from Rabdosia rubescens (a Chinese herbal medicine), could induce ferroptosis by inhibiting γ-glutamyl circulation in YE1 EC cell in vitro [12]. Although research on tumor ferroptosis has been prolific, research on the association between ferroptosis and EC with limited progress made thus far. In this review, we try to summarize important clues about the role of ferroptosis in EC have been found and discuss the future direction of ferroptosis in EC.

## The regulation of ferroptosis

**Fig. 1:**
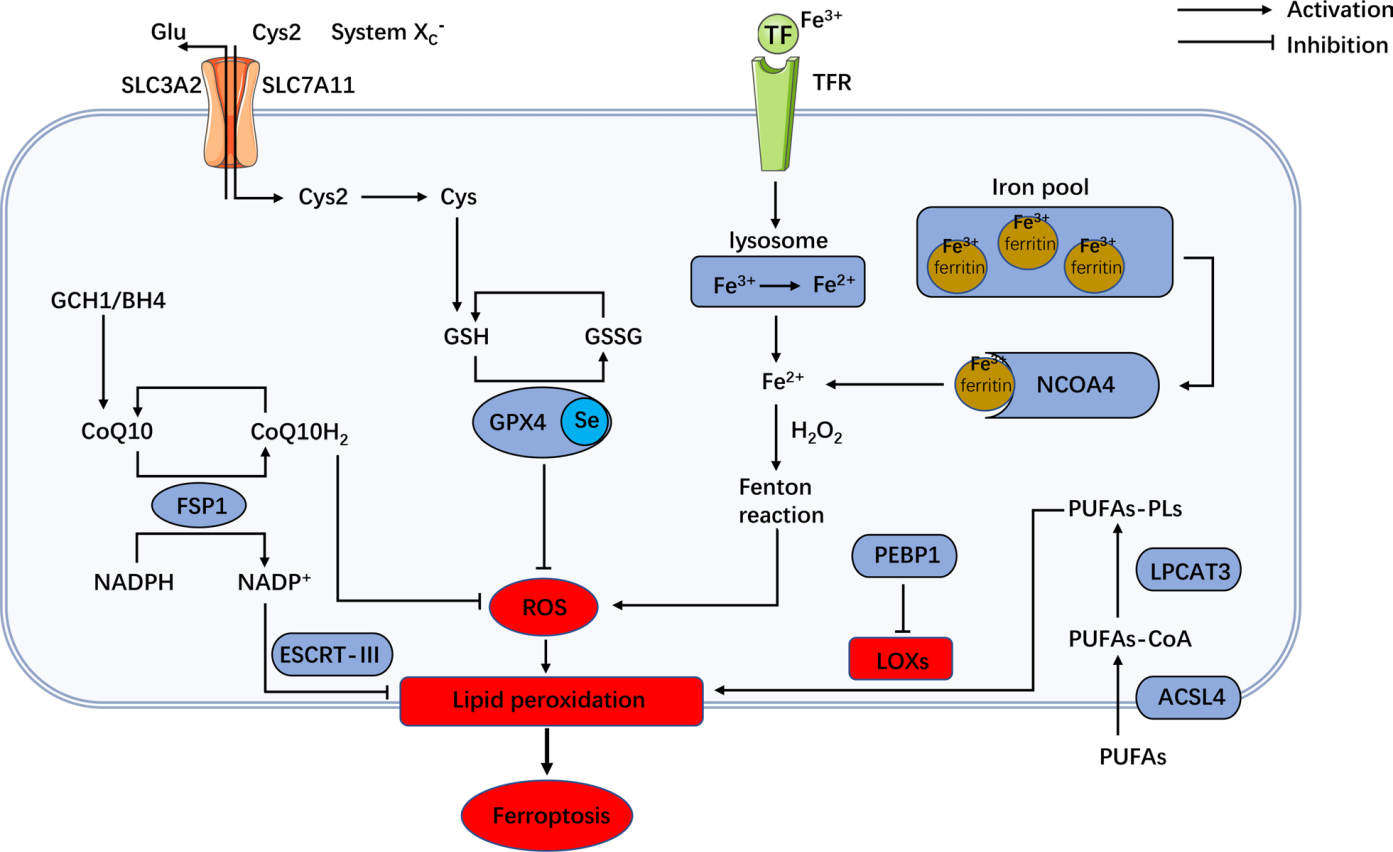
The regulatory mechanism of ferroptosis. Ferroptosis is related to several pathways, including iron metabolism, lipid peroxidation, and GSH-dependent or -independent antioxidant pathways. ACSL4, acyl-CoA synthase long-chain family member 4; BH4, tetrahydrobiopterin; CoQ10, coenzyme Q10; CoQ10H2, reduced form of coenzyme Q10; Cys, cysteine; Cys2, cystine; FSP1, ferroptosis-suppressor-protein 1; GCH1, GTP cyclohydrolase-1; Glu, glutamate; GPX4, glutathione peroxidase-4; GSH, glutathione; GSSG, oxidized glutathione; LOXs, lipoxygenases; LPCAT3, lysophosphatidylcholine acyltransferase 3; NADPH, nicotinamide adenine dinucleotide phosphate; NCOA4, nuclear receptor coactivator 4; PEBP1, phosphatidylethanolamine-binding protein 1; PUFA, polyunsaturated fatty acid; ROS, reactive oxygen species; SLC3A2, solute carrier family 3 member 2; SLC7A11, solute carrier family 7 member 11; System Xc^−^, cysteine/glutamate transport protein system; TF, transferrin; TFR, transferrin receptor

The regulatory mechanism of ferroptosis is correlated with several pathways, including iron metabolism, lipid peroxidation, and glutathione (GSH)-dependent or -independent antioxidant pathways (Fig. 1).

### Iron metabolism

Iron is one of the essential nutrients for living organisms. In general, intracellular iron balance is the regulated through several aspects of iron metabolism, including iron absorption, utilization, output and storage. When ferroptosis occurs, large amounts of free Fe^2+^ are accumulated in the cells. Free Fe^2+^ is highly oxidized and prone to Fenton reaction with hydrogen peroxide (H_2_O_2_), generating hydroxyl radicals which can cause oxidative damage to proteins, DNA and membrane lipids. The reaction between hydroxyl radicals and membrane lipids (especially polyunsaturated fatty acids) leads to lipid peroxidation [13]. The occurrence of lipid peroxidation reaction induce the damage of cell membrane and lead to ferroptotic cell death [14].

Intracellular free iron participates in the biosynthesis of iron sulfur clusters and heme [15]. As an important cofactor in the tricarboxylic acid cycle and mitochondrial respiratory chain, iron is indispensable in many key life processes [16]. Intracellular Fe^2+^ directly participates in Fenton reaction, drives oxygen and redox metabolism and the production of ROS, improves the level of intracellular oxidative stress and promotes the occurrence of ferroptosis. There are two major sources of Fe^2+^: (i) the transferrin (TF) carrying Fe^3+^ binds to the transferrin receptor (TFR) on the plasma membrane and is absorbed into the cells. The hydrogen ion concentration (pH) difference inside and outside the cells makes the bound iron release in the form of Fe^2+^, or combines with ferritin in the form of Fe^3+^ [17, 18]; and (ii) Iron can combine with ferritin. The ferritin in the iron pool can be encapsulated by autolysosome under the mediation of nuclear receptor coactivator 4 (NCOA4) [19], and then degrade and release a large amount of Fe^2+^.

### Lipid peroxidation

Hydrogen atoms in lipids are lost by free radicals or lipid peroxidase in a reaction called lipid peroxidation, resulting in the oxidation, fracture and shortening of lipid carbon chain, and the production of, lipid hydroperoxides and active aldehydes (e.g., malondialdehyde, 4-hydroxynonenal). Polyunsaturated fatty acid (PUFA) has a high affinity with free radicals, and the hydrogen atoms between its double bonds are easily oxidized by free radicals. During the ferroptosis, lipid peroxidation may lead to the oxidation degradation of lipids, such as PUFAs-containing phosphatidylethanolamines (PE). The oxidative degradation of lipids ultimately leads to cell damage [20]. In addition, lipid peroxidation may change the molecular configuration of PUFA, destroy the cell membrane structure, resulting in reduced fluidity and stability, and resulting in increased permeability of cell membrane and ultimately cell death [7].

Studies have shown that lysophosphatidylcholine acyltransferase 3 (LPCAT3) and Acyl-CoA synthase long-chain family member 4(ACSL4) are vital drivers of ferroptosis [8]. ASCL4 plays a key role in ligating long-chain PUFAs with coenzyme A. Then, these products can be re-esterified into phospholipids by some LPCAT enzymes (e.g., LPCAT3) to increase the cellular incorporation of long-chain PUFAs into membranes [8, 21, 22]. Meanwhile, some studies have found that lipoxygenases (LOXs) (especially LOX-15) play the vital role in ferroptosis [23, 24]. Overexpression of LOXs can lead cells more prone to ferroptosis, and the direct oxidation of PUFAs catalyzed by LOXs promotes to the occurrence of ferroptosis [25]. Surprisingly, high expression of 5-lipoxygenase (5-LO) was found in human ESCC tissues and was significantly associated with advanced disease and lymph node metastasis, and 5-LO expression was found to induce cancer cell proliferation in vitro [26]. LOXs inhibitors, such as zileuton, baicalein, AA-861, and CDC, protect cells from GPX4 inhibitor RSL3, demonstrating the critical role of LOXs in cell ferroptosis [23, 27]. Phosphatidylethanolamine-binding protein 1 (PEBP1) prevents the peroxidation of PUFAs by increasing LOX-15 localization at the plasma, thus preventing ferroptosis [28].

### GSH-dependent antioxidant pathway

GSH, a water-soluble tripeptide, is composed of amino acid residues of glutamate, cysteine, and glycine. There are two types of glutathione in the human body, one is reduced GSH and the other is oxidized glutathione (GSSG). GSH is a vital antioxidant in human body. It not only reduces H_2_O_2_ to H_2_O, scavenges free radicals and maintains the equilibrium state of intracellular free radicals, but also serves as a key cofactor of GPX4. GPX4, an antioxidative enzyme, participates in eliminating intracellular lipid ROS, thus preventing the occurrence of ferroptosis. GSH can cooperate with GPX4 to terminates lipid peroxidation reaction, promotes the reduction reaction of lipid peroxides of cell membrane, and antagonizes the induction of ferroptosis. The inactivation of GPX4 caused by GSH depletion which increases intracellular lipid peroxidation, leading to ferroptosis [29].

Ferroptosis inducers RSL3 [29] and ML210 [30] induce ferroptosis by irreversibly binding to, an active selenocysteine site of GPX4. Drugs that are able to downregulate the expression of GPX4 protein can also induce ferroptosis. For example, FIN56 [31] and PdPT [32] induces ferroptosis by promoting the degradation of GPX4 protein or reducing its intracellular protein abundance.

The solute carrier family 3 member 2 (SLC3A2) and solute carrier family 7 member 11 (SLC7A11) constitutes the cysteine/glutamate transport protein system (system X_C_^−^). System X_C_^−^ function depends upon the uptake of cystine and the exchange of intracellular cystine and glutamate at a ratio of 1:1. The light chain subunit SLC7A11 is highly specific to cystine and glutamate and is responsible for the basic transport activity of the system X_C_^−^, while the heavy chain subunit SLC3A2 mainly acts as a companion protein and regulates the transport of SLC7A11 to the plasma membrane [33, 34]. After entering the cell, cystine is rapidly reduced to cystine and used to synthesize GSH [35]. The inhibition of the activity of system X_C_^−^ subunit induces, the insufficient cell intake of cysteine, thus imped the synthesis of GSH. The remaining intracellular GSH is rapidly consumed by H_2_O_2_ or lipid peroxide, and oxidative stress provokes damage to macromolecules, including lipids [36]. At the same time, the decrease of intracellular GSH concentration causes a loss of the activity of GPX4, resulting in a sharp decline in cell survival under lipid peroxidation and ferroptosis.

For example, experimental agent erastin can reduce the entry of extracellular cystine and affect GSH synthesis through targeting inhibition of system X_C_^−^ on the cell surface. GSH depletion leads to inhibition of GPX4 activity and accumulation of lipid ROS, which induces cell ferroptosis [37]. Clinical drugs, such as sorafenib (a Raf inhibitor) and sulfasalazine (an anti-inflammatory drug) can also induce ferroptosis through this mechanism [38].

### GSH-independent pathway

It is worth mentioning that Doll et al. [39] and Bersuker et al. [40] found that apoptosis inducing factor mitochondria-associated 2 (AIFM2) was later renamed ferroptosis-suppressor-protein 1 (FSP1), which can inhibit cell ferroptosis. Also, FSP1 and coenzyme Q10 (CoQ10) are synergistic in the scavenger of lipid peroxidation. FSP1/CoQ10 acts as a parallel system independent of GSH, synergistically inhibiting phospholipid peroxidation and ferroptosis with GPX4 and GSH [41]. Inhibitors of FSP1 and GPX4 can play a synergistic effect to induce ferroptosis in many cancers [39].

Another important mechanism that FSP1 fights lipid peroxidation is through ESCRT-III dependent membrane repair mechanisms, thereby preventing the occurrence of cell ferroptosis [42]. Further, it is speculated that targeting the FSP1-ESCRT-III pathway may enhance the effect of ferroptosis activators in tumor cells, including EC [43]. In EC, the expression of FSP1 was observably increased and significantly associated with the infiltration of CD4^+^T cells. FSP1 may influence the progression of EC by regulating the way ferroptosis occurs in different immune cells [44].

Coincidentally, two independent teams have discovered GTP cyclohydrolase-1 (GCH1)- tetrahydrobiopterin (BH4) pathway protects cancer cells from ferroptosis independent of GSH [45, 46]. GCH1, as a key enzyme for intracellular synthesis of BH4 [47], is a coenzyme of nitric oxide synthase and has a powerful antioxidant effect. GCH1 overexpression protects GPX4-knockout cells from ferroptosis, demonstrating that GCH1 is a completely GPX4-independent pathway [45]. The using of BH2 or BH4 saves cells treated with ferroptosis inducers [46]. In addition, AUF1 (an RNA-binding protein) was positively associated with the expression of GCH1. The inhibition of AUF1 obviously increases the apoptosis of ECA-109 cells, while inhibition of GCH1 inhibits the proliferation of ESCC cells [48]. Thus, it is possible that inhibition of GCH1 leads to a decrease in BH4, which leads to ferroptosis in EC cells. Of course, this hypothesis remains to be verified in future studies, and the regulatory pathway of ferroptosis, GCH1-BH2/BH4, also needs further research.

## Influence of ferroptosis related pathway on esophageal cancer

### Prediction of prognosis of ferroptosis related genes in EAC and ESCC

Zhu et al. [49] examined genes related to ferroptosis in patients with EAC using The Cancer Genome Atlas (TCGA) database, and they found the ferroptosis-related genes were mainly associated with lipid metabolism, iron metabolism, energy metabolism and anti-oxidative metabolism. Cox regression analysis was used to identify four ferroptosis-related genes (CARS1, GCLM, GLS2 and EMC2) and these genes had predictive value for overall survival (OS) of EC. The team further validated these genes in EAC patient tissues and found that GCLM and GLS2 were significantly associated with CD8^+^T cells, suggesting a complex relationship between ferroptosis and immunity.

To provide more comprehensive understanding of immunotherapy for ESCC, Lu et al. [50] screened 45 ferroptosis-related genes based on abnormal gene expression in ESCC, and established a prediction model of ferroptosis-related genes based on the results of Cox regression analysis. They found that patients who got a lower risk score had a higher proportion of CD4^+^ memory active T cells, CD8^+^T cells, and macrophages. They also confirmed that ferroptosis-linked ESCC immune microenvironment influenced patient outcomes to some extent. It is worth noting that SCP2, MAPK, PRKAA1 and other genes screened in this study have been proven to play various roles in the process of cell ferroptosis [51–53].

Liu et al. [54] obtained 18 pairs of differentially expressed ferroptosis-related long non-coding RNAs(DEfalncRNAs)by analyzing tumor samples and normal tissues, and established prognostic characteristic models. Through statistical analysis, they believed that it was possible to predict the survival expectation, immunotherapy effect and drug sensitivity of EC patients, which could contribute to individualized treatment and clinical prediction. At the same time, the function of long-chain noncoding RNA (IncRNA) in ferroptosis and cancer has been elucidated in a growing number of literatures. Seven-IncRNA signature was shown to be a better predictor of patient survival in ESCC than tumor node metastasis classification (TNM) staging alone [55]. The survival time of patients in the low-risk group was obviously higher than that of the high-risk group, which was the same result as Liu’s study. In other cancers, LINC00336 can inhibit ferroptosis and promotes lung cancer cell growth [56], and contribute to ferroptosis in leukemia cells by increasing ROS and iron [57]. Moreover Liang et al. [58] used TCGA and ICGC databases to screen genes associated with ferroptosis in hepatocellular carcinoma patients, and established a prediction model for OS. At the same time, they also observed that ferroptosis was closely associated with the immune process of cancers, but the method used was different from Lu et al.’s [50]. Collectively, these studies have highlighted a potential role of ferroptosis in regulation of immune function. These findings may lead to improved therapeutic approaches for ESCC.

### Potential effects on the p53 pathway

P53, an important cancer suppressor gene, has been observed to be mutated or inactivated in more than half of cancers. P53 gene mutations are quite common in EC, occurring in 40–60% of EC cases, even in the early stages of cancer [59]. The inhibitory effect of p53 on tumor cells mainly depends on the induction of cell cycle stagnation, senescence, or apoptosis. In addition, recent studies have found that it can regulate ferroptosis in cancer by regulating oxidation–reduction state and metabolism [60].

According to the mutation status and cell environment of p53, it has a dual effect in promoting or inhibiting ferroptosis. p53 promotes ferroptosis of tumor cells by inhibiting SLC7A11 transcription and reducing cystine uptake during cell stress. For example, activation of p53 by nutlin-3 triggers ROS-induced stress that cause ferroptosis in osteosarcoma cells [61]. A mutated form of p53, missense mutation, such as p53R273H and p53R175H, block NRF2-mediated upregulation of SLC7A11 and inhibit SLC7A11 expression [62, 63]. In addition to regulating SLC7A11, p53 also regulates ferroptosis sensitivity in tumors by targeting a polyamine metabolism-related gene, spermidine/spermine N-acetyltransferase 1 (SAT1) [64]. One recent study found that radiation-induced p53 activation inhibits the expression of SLC7A11, leading to lipid peroxidation and ferroptosis in EC cells [65].

Under certain conditions, p53 can also negatively regulate ferroptosis. For example, in colorectal cancer, deletion of p53 obstructs the accumulation of dipeptidyl-peptidase-4 (DPP4) in the nucleus, promotes DPP4 and NOX1(NADPH oxidase 1) complex formation, and enhances lipid peroxidation and ferroptosis [66]. In conclusion, the effect of p53 on ferroptosis is dependent on gene mutation and cell type. However, the exact mechanism of p53 regulating ferroptosis in EC needs to be further elucidated.

### Potential effects of the NRF2 pathway

The transcription factor nuclear factor erythroid 2-related factor 2 (NRF2) is considered to be main regulator of antioxidant reaction [67]. The target gene of NRF2 involves the process of regulation of iron metabolism, regulation of exogenous substances and catabolism of reactive aldehydes, GSH synthesis, NADPH regeneration, which participate in regulation of REDOX status in cells [68]. The negative expression of NRF2 in ESCC biopsy specimens was associated not only with good efficacy of chemoradiation therapy (CRT), but also with a better prognosis of ESCC [69]. In contrast, NRF2 was overexpressed in ESCC, which predicted poor prognosis of patients [70, 71]. NRF2 expression in ESCC tissues and cells was significantly up-regulated as indicated by immunohistochemical staining [72]. Meanwhile, NRF2 can promote autophagy by activating Ca^2+^/calmodulin‑dependent protein kinase II α (CaMKII α) to enhance the radiation resistance of ESCC [73]. Animal experiments also showed that Polygalacin D (a Chinese herbal medicine extract) could inhibit tumor growth in ESCC mouse model through miR-142-5p /NRF2 axis [72]. A study showed that neferine, an anticancer active substance extracted from *Nelumbo Nucifera*(Lotus), inhibited the growth of ESCC by inhibiting NRF2 expression and promoting ROS production to induce apoptosis [74]. To summarize, these observations suggest a pro-tumorigenic role for NRF2 in ESCC.

The expression level of NRF2 evidently affects the sensitivity of cancer cells to ferroptosis, because the high expression of NRF2 can help cancer cells resist ferroptosis, while reducing NRF2 content can increase the sensitivity of cells to ferroptosis inducers [75, 76]. ARF was identified as a vital regulator of NRF2 by biochemical purification. The research team reported that NRF2-ARF interactions play a vital role in the non-P53-dependent ferroptosis response in human cancer cells [77]. At present, a number of relevant studies have also mentioned that high expression of NRF2 in other cancer cells (such as lung cancer) can promote cancer progression and metastasis, and also play a role in making tumor cells resistant to radiotherapy and chemotherapy [78–81]. NRF2-mediated SLC7A11 might enhance therapeutic resistance by inhibiting ferroptosis through experiments. In ESCC patients, high levels of NRF2 and SLC7A11 were related with low progression‑free survival (PFS), OS, and poor treatment response [82].

NRF2 target genes are involved in broad antioxidant function, iron metabolism and metabolites of intermediate cell metabolism [83]. For example, both GPX4 and SLC7A11, two of the most important anti-ferroptotic genes, are regulated by NRF2 [84]. However, the genes responsible for the anti-ferroptotic role of NRF2 in ESCC remain to be identified.

### Potential effects of the Hippo-YAP pathway

The Hippo pathway regulates gene expression to affect changes in cell shape, density, and adhesion, and its dysfunctions are often associated with squamous cell carcinoma (SCC), including ESCC [85]. Yes-associated protein (YAP), a key downstream transcription factor of Hippo pathway, plays a role in regulating cell growth, proliferation and apoptosis [86]. Overexpression of YAP is often found in ESCC and it is positively correlated with histological stage and grade of ESCC as well as OS and PFS of patients [87]. Similarly, Zhao et al. reported that YAP1 down-regulation significantly inhibited EC cell invasion and in vitro and vivo [88].

Studies have confirmed that ferroptosis, to some extent, depends on cell density, and Hippo signaling pathway, which can be driven by tumor suppressor NF2 [89]. Activation of NF2 can down-regulate E3 ubiquitin ligase CRL4^DCAF1^ and inhibit the degradation of Lats1/2 in Hippo pathway [90]. This further promotes the phosphorylation of YAP, which limits its nuclear location. YAP-mediated expression of transferrin receptor 1(TFRC) and ACSL4 are key players in determining ferroptosis sensitivity [89]. The upregulation of PARK2 can promote the degradation of YAP in ESCC cells, and inhibit the activation of Hippo-YAP pathway and the progression of ESCC [91]. Therefore, regulation of Hippo-YAP pathway activity suggests potential for regulating ferroptosis in EC.

### Potential effects of DNAJB6

The DnaJ (heat shock protein 40 family protein) homolog, subfamily B, member 6 (DNAJB6) belongs to the HSP40/DNAJ chaperone family [92, 93]. The aggregation of DNAJB6 protein is commonly found in neurological diseases such as Parkinson's disease and Huntington's disease [94]. Yu et al. observed high expression of DNAJB6 in ovarian cancer tissues, and speculated that DNAJB6 could be a potential target for patient prognosis [95]. DNAJB6a has also been found to inhibit the progression of breast cancer cells [96]. There are several studies on the role of DNAJB6 in EC.

As a major oncogene, AKT abnormal activation is usually mediated by AKT1 phosphorylation [97]. DNAJB6a in EC cells could regulate and inhibit tumor cell proliferation through AKT1, thus playing a role in cancer suppression [98]. In recent studies, Jiang et al. found that GPX4 level was down-regulated in DNAJB6a overexpressed ESCC cells, accompanied by smaller mitochondria, increased membrane density, loss of mitochondrial structure integrity and edema of mitochondrial matrix, which were typical characteristics of ferroptosis. Therefore, they concluded that overexpression of DNAJB6a promoted ferroptosis in ESCC cells. At the same time, lymph node metastasis was more common in ESCC patients with low DNAJB6 levels than patients with high DNAJB6 levels [11]. However, relevant studies are still insufficient, and the explicit mechanism of how DNAJB6 causes ferroptosis in cells remains to be further explored.

## Ferroptosis in the treatment of esophageal cancer

### Potential therapeutic effect of SLC7A11 inhibitor on esophageal cancer

According to recent studies, SLC7A11 has considerable potential as a cancer therapeutic target [99]. The ideal therapeutic target for anticancer drugs should be specifically selective for cancer growth, with drugs that can produce the desired toxic killing effect in cancer cells with little or no unnecessary side effects on normal tissue. The SLC7A11 seems to fit those criteria. Because high levels of oxidative stress often occur in cancer cells [100], which have higher antioxidant defense requirements. Therefore, cancer cells rely more on SLC7A11 than normal tissue to acquire cysteine and maintain redox steady state, which is very similar to oncogene dependence in cancer development. Several studies have shown that SLC7A11 promotes drug resistance and radiotherapy resistance by inhibiting ferroptosis in cells [99, 101–103]. Meanwhile, in ESCC patients, high expression of SLC7A11 was related to low PFS, OS and poor treatment response [82].

At present, some compounds have been validated and identified as SLC7A11 inhibitors, including the most classic ferroptosis inducer erastin, and some other compound discovered in recent years, such as sulfasalazine, cisplatin, sorafenib, and artesunate [104–106]. Sulfasalazine could inhibit the progression of EC cells in a dose-dependent manner in vitro, and suppress the colony formation of tumor cells [107]. SLC7A11 has also been demonstrated as a potential therapeutic target and sulfasalazine in various other cancer, including small-cell lung cancer [108], head and neck cancer [109], hepatocellular carcinoma [110] and urogenital cancer [111]. Cisplatin is universally used in the treatment of advanced nonoperative EC due to its remarkable anticancer activity [112, 113]. According to Guo et al. [114], combination of erastin and cisplatin enhanced the antitumor effect of cisplatin. They suggest that cisplatin induces ferroptosis is mainly due to direct intracellular consumption of GSH. This is similar to the conclusion of Roh et al. [109]. Up to now, there is no denying that SLC7A11 is a very potential target that needs to be further studied, but efficient and specific drugs for use in the clinic are still lacking.

### Potential therapeutic effects of GPX4 inhibitors on esophageal cancer

Currently known inhibitors of GPX4 mainly include RSL3, ML210, Withaferin A, and some diverse pharmacological inhibitor (DPI) compounds, which have been shown a good anti-cancer activity in a series of cancers [104, 115]. These agents covalently bind to the active selenocysteine site of GPX4 and inhibit its enzyme activity, resulting in reduced lipid repair ability, accumulation of lipid peroxides, and ultimately intracellular ferroptosis [23]. One recent study showed that 5-aminolevulinic acid (5-ALA) significantly inhibited GPX4 in esophageal cancer KYSE30 cells, resulting in ferroptosis. Therefore, they speculated that 5-ALA could exert anti-tumor effect through induction of ferroptosis [116].

When targeted therapy or chemotherapy agents are combined with GPX4 inhibitors, they can effectively reduce resistant cancer cells. However, combination therapy with multiple agents increases the probability of adverse effects, compromising patient safety and treatment outcomes [115]. Unfortunately, although GPX4 inhibitors can achieve certain therapeutic effects in vitro, their low solubility and poor pharmacokinetic properties prevent their use in vivo. Given the current promise of ferroptosis in the treatment of drug-resistant tumors, it is imperative to develop an effective bioavailable inhibitor of GPX4.

### Therapeutic effect of oridonin-induced ferroptosis on esophageal cancer

Diterpenoids are compounds extracted from plants with a series of complex pharmacological effects such as anti-inflammatory, antibacterial, antioxidant, and anticancer. Some of diterpenoids have been shown to have anticancer effects on EC in vitro [117]. As a widely used diterpenoid compound, oridonin has also received much attention for its antitumor effects [118]. Oridonin significantly induced hepatic stellate cells apoptosis and triggered GSH depletion in the hepatic stellate cells [119]. Zhang et al. reported that oridonin-treated EC cells showed lipid peroxidation, cell proliferation inhibition, and cell death. And the process can be blocked by specific ferroptosis inhibitors, including hepatic stellate cells [12]. They suggested that oridonin induced cell ferroptosis primarily by affecting the γ -glutamate cycle, thus achieving antitumor effects [120]. Ferroptosis induced by oridonin in tumor cells has also been observed in vitro. However, the clinical effects of oridonin need to be further studied.

### Potential role of ferroptosis in chemoradiotherapy

A significant number of EC patients are initially diagnosed as advanced stage, accompanied by local or distant metastasis. The existing main treatment methods, including neoadjuvant chemotherapy or radiotherapy and chemotherapy combined with surgical treatment, can bring patients better survival expectations than traditional surgery, but some patients still cannot achieve the expected treatment effect [121, 122]. The main factor affecting the therapeutic effect is multidrug resistance(MDR) of cancer [123].

It has been suggested that regulation of intracellular ROS levels can sensitize MDR cancer cells to certain chemotherapeutic drugs, thus promoting the death of MDR cancer cells [124, 125]. Cisplatin induced MDR in tongue squamous cell carcinoma by up-regulating the expression of SLC7A11 in NRF2 and ATF4-dependent manner, thereby interfering the expression of SLC7A11 and promoting the anti-cancer efficacy of cisplatin [126]. GPX4 inhibitor RSL3 can enhance the anti-tumor effect of cisplatin by increasing the accumulation of ROS and labile iron pool (LIP) levels [127, 128]. Meanwhile, inhibition of NRF2 was found to reverse resistance to RSL3-induced ferroptosis [129]. Given that interfering the expression of ferroptosis-related genes or using ferroptosis inducers can enhance the effect of radiotherapy and chemotherapy, ferroptosis-based strategies may lead to different possibilities for the treatment of EC.

### Potential role of ferroptosis in immunotherapy

Immunotherapy is a research hotspot in tumor field and has shown encouraging clinical efficacy. Among them, immune checkpoint inhibitors, as the focus of immunotherapy research, has had a profound impact on the treatment of various tumors and changed the traditional treatment of EC. Clinical trials KEYNOTE-180 and ATTRAVTION-1 showed that immune checkpoint inhibitors pembrolizumab and nivolumab had good efficacy in patients with metastatic EC [130, 131].

**Fig. 2:**
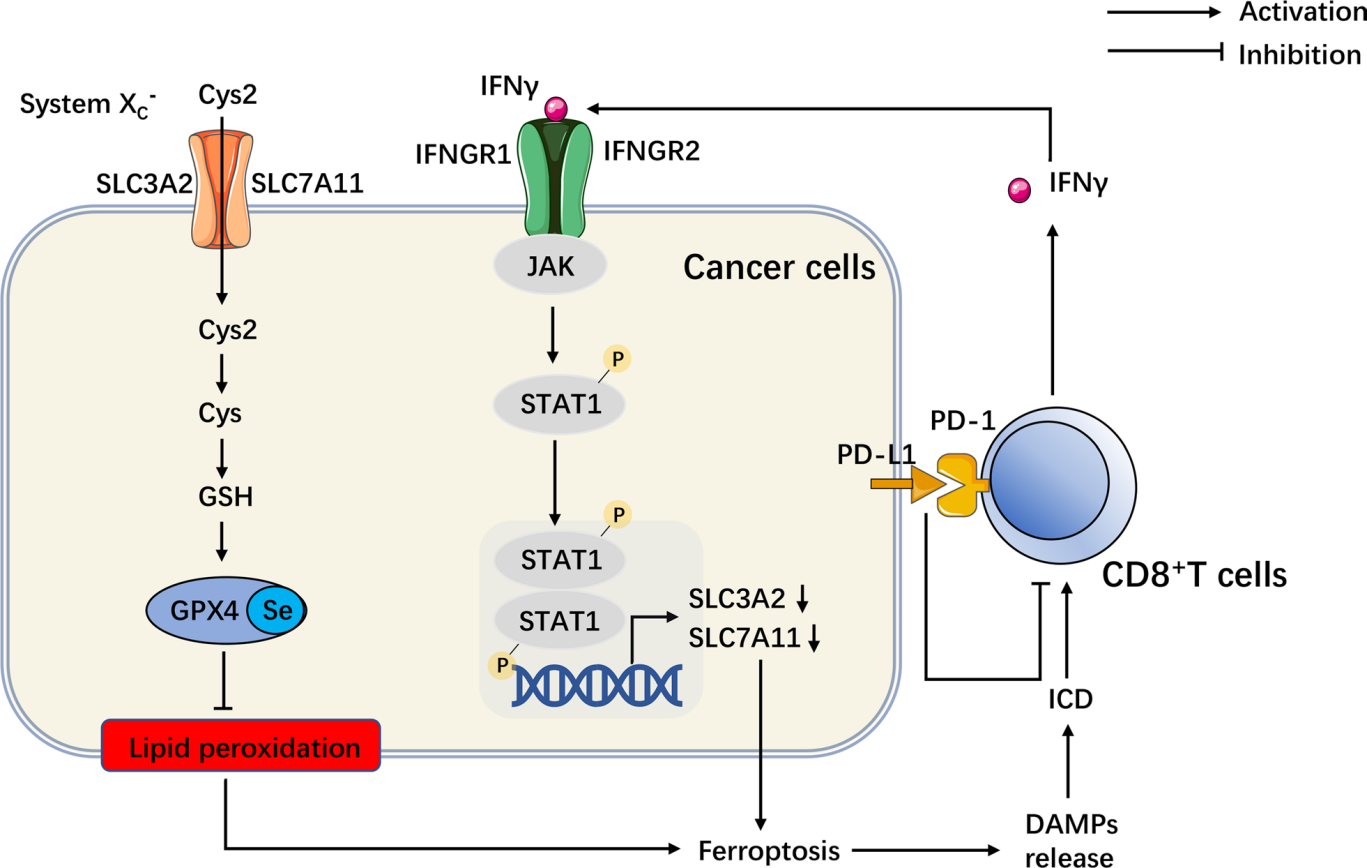
Potential role of ferroptosis in immunotherapy. CD8+ T cells release IFNγ, activate JAK-STAT1 pathway, down-regulate SLC3A2 and SLC7A11, and reduce cystine uptake by cancer cells, resulting in cell ferroptosis. Cys, cysteine; Cys2, cystine; DAMPs, damage-associated molecular patterns; GPX4, glutathione peroxidase-4; GSH, glutathione; ICD, immunogenic cell death; IFN γ, interferon γ; JAK, janus kinase; PD-1, programmed death-1; PD-L1, programmed death-ligand 1; Se, selenium; SLC3A2, solute carrier family 3 member 2; SLC7A11, solute carrier family 7 member 11; STAT1, signal transducer and activator of transcription 1; System Xc^−^, cysteine/glutamate transport protein system

Cytotoxic T lymphocyte associated protein 4 (CTLA-4), programmed death-1 (PD1) and programmed death-ligand 1 (PD-L1) are the most representative immune checkpoint pathways. Their role is to prevent autoimmune diseases caused by excessive activation of immune response, the combination of PD-L1 with PD-1 on the surface of immune cells can reversibly inhibit the activation and proliferation of T cells and produce immunosuppression. A recent study found that blocking PD-L1 and CTLA-4 can inhibit the growth of melanoma in animal models by inducing ferroptosis [132]. CD8^+^T cells activated by immunotherapy down-regulate SLC3A2 and SLC7A11 by releasing interferon γ (IFN γ), which activates the JAK-STAT1 pathway, reduces cystine uptake by cancer cells, and promotes ferroptosis. The induction of ferroptosis in turn synergically enhances the anti-cancer effect mediated by T cells. Ferroptosis may be the key to coordinate cancer immunotherapy with conventional chemoradiotherapy [132] (Fig. 2). Efimova et al. first reported that ferroptosis can induce immunogenic cell death both in vitro and in vivo. The release of damage-associated molecular patterns (DAMPs), especially ATP and high-mobility group box 1 (HMGB1), inhibits cancer cell growth by promoting ferroptotic immunogenic cell death [133] (Fig. 2).

Despite extensive use of immunotherapy in other malignancies, the number of approved immunotherapies for gastrointestinal tumors remains limited [134]. For EC, immunotherapy only as a second- or third-line treatment [135, 136]. Ferroptosis immunotherapy for EC still has considerable development prospects. Immunotherapy-mediated regulation of ferroptosis in cancer cells has great potential, but its related mechanisms are complex and there are still many uncharted areas that deserve further exploration. The possibility of ferroptosis immunotherapy for EC needs to be validated in animal models and ultimately used in the clinic.

## Conclusion and perspective

Ferroptosis, as a non-apoptotic programmed cell death mediated by iron, is initiated and executed under strict molecular regulation mechanism. Although many studies have confirmed the close relationship between ferroptosis and various diseases, the role of ferroptosis in EC is still in its infancy. At present, the research of ferroptosis in the field of EC is still focused on using genetic data from the database to screen ferroptosis-related genes to predict the prognosis of patients with EC [49, 50, 54]. At the same time, these findings are helpful for patients to judge immunotherapy and drug sensitivity, and indicate that the immune process of EC is highly correlated with ferroptosis, which may be a key direction of future research.

As an adjuvant therapy for ferroptosis inducer, cancer therapy based on the molecular regulation mechanism of ferroptosis has great development potential. However, ferroptosis is a double-edged sword. The potential toxic and side effects of inhibitors or inducers of key pathways in ferroptosis should be fully studied to determine that they can specifically trigger Fenton reaction in cancer cells and avoid off-target toxicity to normal cells causing cancer or other diseases. Currently most of ferroptosis inducers currently in use are targeting SLC7A11 or GPX4. Only a few ferroptosis inducers, such as sulfasalazine, altretamine [137] and sorafenib, have been approved for use by the Food and Drug Administration (FDA), but these drugs are not included in the guidelines for standard treatment of EC. Meanwhile, known ferroptosis inducers such as RSL3 and Withaferin A cannot be used in clinical trials due to pharmacokinetic and non-specific reasons, and are only used for laboratory studies [104]. Two studies using Oridonin and 5-ALA to treat EC cells found these two agents induced ferroptosis, but the exact mechanism remains to be further explored. In addition, it is worth to investigate the potential role of ferroptosis inducers or inhibitors that targeting GSH-independent pathway in ESCC. For example, the recently reported ferroptosis-related FSP1-ESCRT-III pathway and GCH1-BH4 pathway may provide new targets and ideas for EC therapy.

Immunotherapy is the focus of anti-cancer research in recent years, and many studies have elaborated the complex relationship between immune system and ferroptosis. Ferroptosis may be the key to coordinate cancer immunotherapy with conventional chemoradiotherapy. Regulation of ferroptosis is of great significance for reasonable and effective integration of immunotherapy and chemoradiotherapy. It should not be ignored that cancer (including EC) is a heterogeneous disease, and personalized medicine will be the focus of future research. The basic research and clinical transformation of ferroptosis still have many unknown and challenges. Future research on inducing ferroptosis will certainly help patients with EC and other diseases.

## Data Availability

Not applicable.
